# Preliminary experience of Tubridge Plus flow diverter in the treatment of intracranial aneurysms

**DOI:** 10.3389/fneur.2026.1862240

**Published:** 2026-07-09

**Authors:** Tao Zhang, Xing Guo, Yufeng Liu, Guoqiang Luo, Wei Fang, Hu Chen, Haiyang Jiang, Zijian Yang, Yue Si, Dayun Feng, Zhenwei Zhao, Jianping Deng

**Affiliations:** Department of Neurosurgery, Tangdu Hospital, The Fourth Military Medical University, Xi’an, China

**Keywords:** endovascular therapy, flow diverter, intracranial aneurysms, prospective study, Tubridge Plus

## Abstract

**Objective:**

The Tubridge Plus flow diverter (TPFD) is an iterative evolution of the previous Tubridge flow diverter (TFD) with enhanced technical features intended to overcome prior limitations. This single-center prospective study reported our preliminary experience using the TPFD for intracranial aneurysms (IAs), with evaluation of its technical feasibility, efficacy, safety, and outcomes.

**Methods:**

Between May and July 2023, 10 consecutive patients with 10 IAs were enrolled and treated with TPFD in our institution. Technical success, intraoperative adverse events, angiographic outcomes were collected and analyzed.

**Results:**

Technical success rate for TPFD delivery and deployment was 100%. Intraoperative adverse events, consisting of distal malapposition and iatrogenic proximal malapposition, occurred in 2 patients (20%), both successfully rescued with adjunctive stenting. No perioperative complications occurred, and only 1 patient (10%) experienced transient postoperative headache as a result of mass effect. Angiographic follow-up (FU) demonstrated that complete occlusion rates (OKM D at 6 months; RROC I at 1 year and last FU) improved progressively over time: 60% at 6 months, 80% at 1 year, and 90% at the last FU, with adequate occlusion rates of 90 and 100% at 1-year and the last FU, respectively. No in-stent stenosis or ipsilateral stroke was recorded in any patient during the entire FU period.

**Conclusion:**

This prospective single-center preliminary experience in 10 patients suggests that TPFD is technically feasible, safe, and effective for the endovascular treatment of unruptured ICA aneurysms. However, given the small sample size and single-center design, further large-scale, multicenter studies with long-term FU are warranted to validate its safety and efficacy as a next-generation flow diverter.

## Introduction

Intracranial aneurysms (IAs) are potentially life-threatening vascular lesions. Unruptured IAs carry a persistent risk of subarachnoid hemorrhage following rupture, which is associated with substantial morbidity and mortality (1–3). The management of IAs, especially those with complex morphology, remains a considerable clinical challenge. Over the past two decades, the introduction of flow diverters (FDs) has revolutionized the endovascular management of IAs. Representing a paradigm shift from intrasaccular embolization to parent artery reconstruction as the primary therapeutic mechanism, FDs have made many previously failed and difficult-to-treat IAs now curable via two main mechanisms: (1) by attenuating blood flow into the aneurysm sac which lead to intrasaccular stasis and thrombosis, and (2) by promoting the formation of a neointimal endothelial layer across the aneurysm neck (4–6).

As the first domestically developed and approved flow diverter (FD) for clinical use in China, the Tubridge flow diverter (TFD) has been widely validated for its reliable efficacy and safety in the treatment of IAs (7–9). Building on this foundation, the Tubridge Plus flow diverter (TPFD) was developed with iterative design enhancements to address limitations of the original TFD. These improvements include full-body radiopacity, optimized flared end structure, increased braiding density, specialized surface modification, and compatibility with a smaller-profile delivery microcatheter. Collectively, these features are hypothesized to improve deliverability, wall apposition, flow diversion efficacy, and biocompatibility relative to the predecessor device.

Despite these theoretical advantages, clinical evidence supporting the use of TPFD in IAs treatment remains lacking. To date, no peer-reviewed study has reported the clinical performance of the TPFD. Therefore, this prospective single-center study aimed to present our preliminary experience and evaluate the technical feasibility, procedural safety, complication profile, and angiographic outcomes of the TPFD for IAs.

## Materials and methods

### Patient selection

This prospective study was approved by the Ethics Committee of Tangdu Hospital. Written informed consent was obtained from all patients prior to enrollment.

The inclusion criteria for the patients with IAs treated by TPFD were as follows: (1) Aged 18–75 years old; (2) diagnosis of IAs in the internal carotid artery (ICA); (3) aneurysm neck ≥ 4 mm or dome/neck ratio < 2; (4) parent artery diameter ranging from 2.0 mm to 6.5 mm; (5) availability of complete clinical, procedural, and follow-up (FU) data.

The exclusion criteria included: (1) Aneurysm associated with arteriovenous malformation (AVM), moyamoya disease (MMD) or other complex vascular lesions; (2) ruptured aneurysm within 30 days; (3) multiple aneurysms within the same arterial territory; (4) parent artery stenosis > 50%; (5) recurrent aneurysm previously treated with stent or stent-assisted coiling; (6) any medical or anatomical condition deemed to interfere with safe device deployment; (7) patients considered unsuitable for study participation by the researchers.

Between May 2023 and July 2023, 10 patients with 10 IAs were enrolled. Patient demographics, aneurysm characteristics, procedural details, and angiographic FU data were recorded and analyzed.

### Tubridge Plus flow diverter

**Figure 1:**
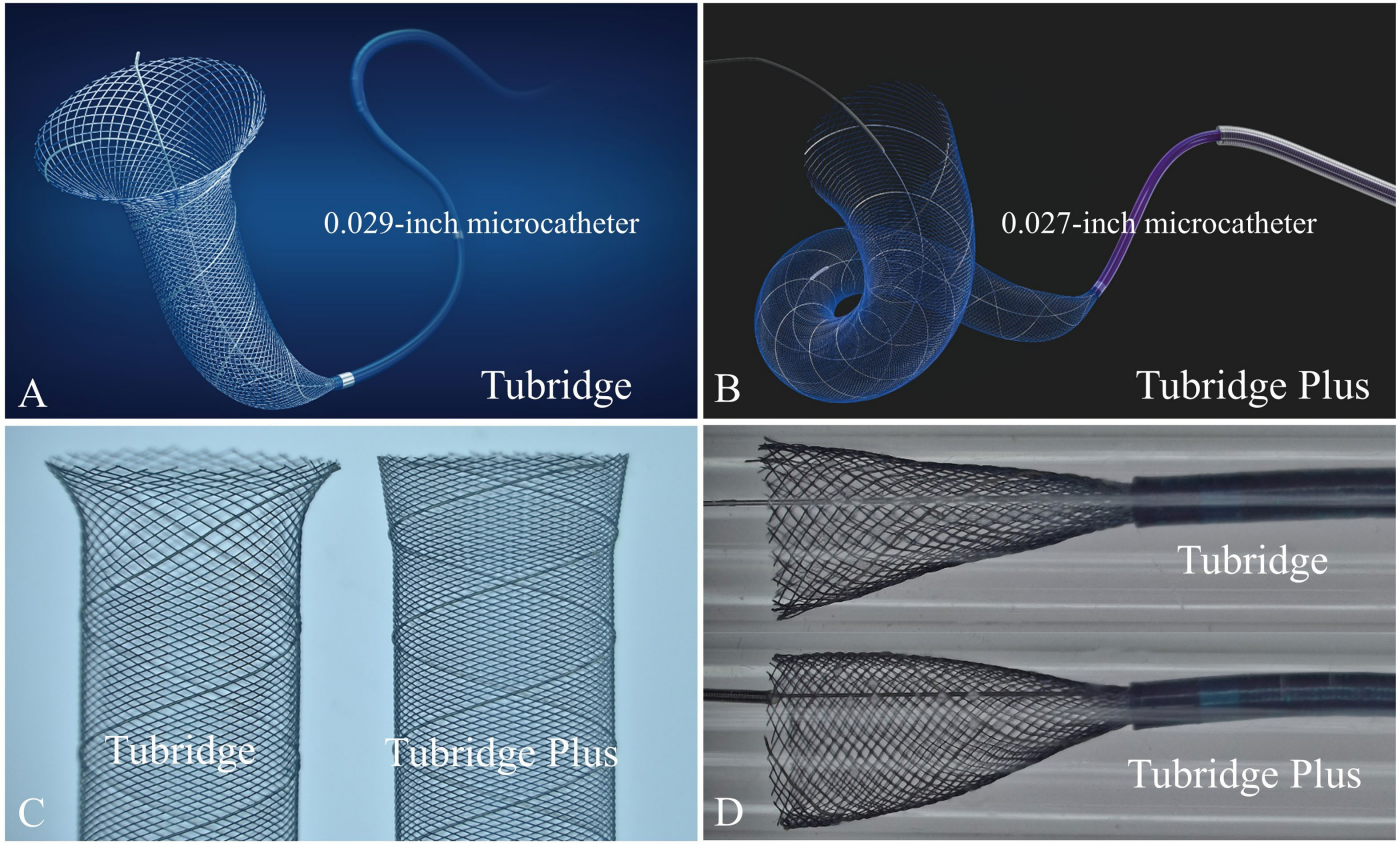
Design comparison of Tubridge and Tubridge Plus. **(A)** Tubridge: compatibility with 0.029-inch microcatheter. **(B)** Tubridge Plus: compatibility with 0.027-inch microcatheter. **(C)** The differences of flare end structure and braiding density between Tubridge and Tubridge Plus. **(D)** The differences of distal configuration during partial deployment from the microcatheter between Tubridge and Tubridge Plus. Reproduced with permission from the copyright holder, MicroPort Medical (China) Co., Ltd.

The TPFD is an upgraded iteration of the TFD, maintaining the fundamental braided stent design while incorporating several key enhancements: (1) Radiopacity: Constructed with nitinol drawn-filled tube (DFT) wires containing a platinum-iridium core, enabling full-body radiopacity and improved intraprocedural visualization; (2) flared ends: Optimized flared geometry with a more gradual flare angle at the ends to improve vessel wall apposition and enhance anchoring stability; (3) braiding density: Increased mesh density for improved flow diversion and accelerated aneurysm thrombosis; (4) surface technology: Integration of BlueSilk Surface^®^ technology to reduce thrombogenicity and promote endothelialization; (5) delivery system: Compatible with a 0.027-inch microcatheter, facilitating navigation in tortuous intracranial vasculature compared with the original 0.029-inch system (Figure 1).

### Endovascular procedures

All endovascular procedures were performed under general anesthesia using the Siemens Artis Zee biplane floor system (Siemens, Munich, Germany) and intravenous heparinization was monitored with an activated clotting time of 250–300 s. A triaxial access system was used for all cases.

An 8Fr Envoy guiding catheter (Codman, Raynham, MA, USA) or a 6Fr NeuroMax long sheath (Penumbra, Alameda, CA, USA) was positioned in the proximal cervical segment of ICA. A 6F (TPFD with coils) or 5F (TPFD only) Navien Intracranial Support Catheter (Covidien, Mansfield, MA, USA) was advanced to the corresponding petrous or cavernous segment of ICA. A Fastrack microcatheter (MicroPort, Shanghai, China) was navigated distal to the aneurysm using a 0.014-inch Synchro microguidewire (Stryker, Kalamazoo, MI, USA). In patients undergoing combined coiling, an additional Echelon microcatheter (Covidien, Mansfield, MA, USA) was selectively catheterized into the aneurysm sac.

Deployment technique for the TPFD was consistent with standard TFD protocols. Post-deployment microguidewire looping and massage were routinely performed to improve wall apposition. In cases of malapposition, an adjunctive Neuroform EZ (Stryker, Kalamazoo, MI, USA) or Solitaire (Covidien, Mansfield, MA, USA) stent was implanted for rescue.

### Antithrombotic regimen

All patients received dual antiplatelet therapy (DAPT) preoperatively: aspirin 100 mg daily and clopidogrel 75 mg daily for 3–5 consecutive days. In patients with clopidogrel resistance, ticagrelor 90 mg twice daily was substituted.

Intra-arterial tirofiban was administered immediately after TPFD deployment as an initial bolus of 10 μg/kg within 3 min, followed by infusion at 0.15 μg/kg/min for 6–12 h. DAPT was resumed 6 h before tirofiban withdrawal. All patients were instructed to take DAPT for 6 months and aspirin for at least 1 year.

### Efficacy and safety outcomes

The efficacy endpoints included: (1) Complete occlusion rate of aneurysms; (2) adequate occlusion rate of aneurysms; and (3) technical success rate of FD delivery and deployment.

The safety endpoints included: (1) Intraoperative and perioperative complications; (2) ipsilateral stroke of target artery; (3) in-stent stenosis or occlusion; and (4) device-related adverse events.

### Clinical and angiographic follow-up

According to the study protocol, Digital subtraction angiography (DSA) was mandatory for all patients at the 6-month FU. For the 1-year and final FU, DSA was strongly encouraged; however, computed tomography angiography (CTA) was accepted for patients who were unwilling or unable to undergo repeat DSA. The potential limitation of CTA in evaluating aneurysm healing after flow diversion, including the risk of metal artifacts that may overestimate residual filling, is acknowledged in the Limitations section.

### Aneurysm occlusion grading

Aneurysm occlusion was graded as follows. At the 6-month FU, when all patients underwent DSA, the O’Kelly Marotta (OKM) scale was used to better characterize the hemodynamic changes and intra-aneurysmal flow patterns induced by the flow diverter at this intermediate time point (10). For the 1-year and final FU, given that a mixture of DSA and CTA was employed, the Raymond-Roy Occlusion Classification (RROC) was adopted to uniformly assess aneurysm healing status across both imaging modalities (11).

Aneurysm occlusion status included: (1) Complete occlusion (OKM D at 6 months; RROC I at 1 year and last FU); (2) Residual neck (OKM C or RROC II); and (3) Residual aneurysm (OKM A/B or RROC III). Adequate occlusion was defined as complete occlusion or residual neck.

## Results

### Patient and aneurysm characteristics

A total of 10 patients (8 females, 2 males) with a mean age of 53.40 ± 11.25 years (range, 41–69 years) were enrolled. All aneurysms were unruptured and incidentally discovered. Five patients presented with dizziness, and one had a history of remote intraparenchymal hemorrhage (IPH).

Nine saccular aneurysms were located at the ophthalmic segment of ICA, and one dissecting aneurysm at the cavernous segment of ICA, according to the Bouthillier classification (12). Among ophthalmic aneurysms, 5 were superiorly oriented and 4 medially oriented.

The mean aneurysm size was 8.67 ± 4.52 mm (range, 3.56–17.7 mm), including 4 large aneurysms (10–25 mm) and 6 small to medium aneurysms (<10 mm). The mean neck width was 5.78 ± 3.13 mm (range, 2.80–11.50 mm), with a mean dome-to-neck ratio of 1.55 ± 0.38 (range, 0.95–1.88). The mean proximal parent artery diameter was 4.86 ± 0.73 mm (range, 3.74–5.99 mm), and the mean distal parent artery diameter was 3.54 ± 0.48 mm (range, 2.80–4.30 mm) (Table 1).

**Table 1:** Patient and aneurysm characteristics treated by Tubridge Plus flow diverter.

Variable	N or mean ± SD
**Sex**	
Male	2
Female	8
**Age (years)**	53.40 ± 11.25
**Presentation**	
Incidental findings	10
Dizziness	5
Previous intraparenchymal hemorrhage	1
**Characteristics of Aneurysm**	
Aneurysm type	
Saccular	9
Dissecting	1
Aneurysm location	
Ophthalmic segment of ICA	9
Superiorly oriented	5
Medially oriented	4
Cavernous segment of ICA	1
Aneurysm size (mm)	8.67 ± 4.52
Small to Medium (<10 mm)	6
Large (10-25 mm)	4
Neck width (mm)	5.78 ± 3.13
Dome-to-neck ratio	1.55 ± 0.38
**Parent artery diameter (mm)**	
Proximal to the neck	4.86 ± 0.73
Distal to the neck	3.54 ± 0.48

ICA, internal carotid artery.

### Procedural outcomes

Technical success of TPFD delivery and deployment was achieved in all 10 patients (100%). Treatment strategy was individualized based on the morphological characteristics of each aneurysm. Large superiorly oriented ophthalmic aneurysms (*n* = 2) were treated with TPFD plus coiling to reduce rupture risk. The remaining aneurysms (*n* = 8) were treated with TPFD alone (Table 2).

**Table 2:** Perioperative and follow-up results of the 10 patients treated by Tubridge Plus flow diverter.

Variable	N or mean ± SD	%
**Technical success**	10	
**Treatment strategy**		
TPD alone	8	80
TPD + Coils	2	20
**Intraoperative adverse events**		
Neuroform EZ for distal malapposition	1	10
Solitaire for proximal malapposition	1	10
**Immediate post-operative angiographic result**		
OKM grading scale A	8	80
OKM grading scale B	1	10
OKM grading scale C	1	10
**Intra-operative complications**	0	0
**Morbidity and mortality**	0	0
**Post-operative symptoms**		
Transient headache for 3 days	1	10
**6-month angiographic follow-up (DSA)**		
OKM grading scale A	2	20
OKM grading scale B	2	20
OKM grading scale D	6	60
Adequate occlusion	6	60
**1-year angiographic follow-up (DSA or CTA)**		
Complete occlusion	8	80
Residual neck	1	10
Residual aneurysm	1	10
Adequate occlusion	9	90
**Last follow-up (DSA or CTA)**		
Months	29.40±0.97	
Complete occlusion	9	90
Residual neck	1	10
Adequate occlusion	10	100
In-stent stenosis	0	0
mRS score = 0	10	100

OKM, the O’Kelly-Marotta grading scale. Reproduced with permission from the copyright holder, MicroPort Medical (China) Co., Ltd.

Intraoperative adverse events occurred in 2 patients (20%), both consisting of device malapposition. Importantly, both events were attributable to procedural technique rather than device failure: (1) Distal malapposition occurred along the concave surface of the parent artery due to suboptimal landing zone selection across a vascular curvature, and was successfully remedied by implantation of an adjunctive Neuroform EZ 4.5 × 20 mm stent. (2) Iatrogenic proximal malapposition resulted from inadvertent dislodgement of the proximal TPFD end during recapture of the Fastrack microcatheter after an otherwise successful initial deployment. A salvage procedure was subsequently performed by implanting an additional Solitaire 6.0 × 30 mm stent. Neither event led to clinical sequelae.

Immediate postoperative angiography showed OKM A in 8 patients (80%), OKM B in 1 (10%), and OKM C in 1 (10%). No intraoperative complications were observed in any patient, and there was no procedure-related morbidity or mortality. One patient (10%) experienced transient postoperative headache lasting 3 days as a result of mass effect, which resolved spontaneously.

### Follow-up outcome

**Figure 2:**
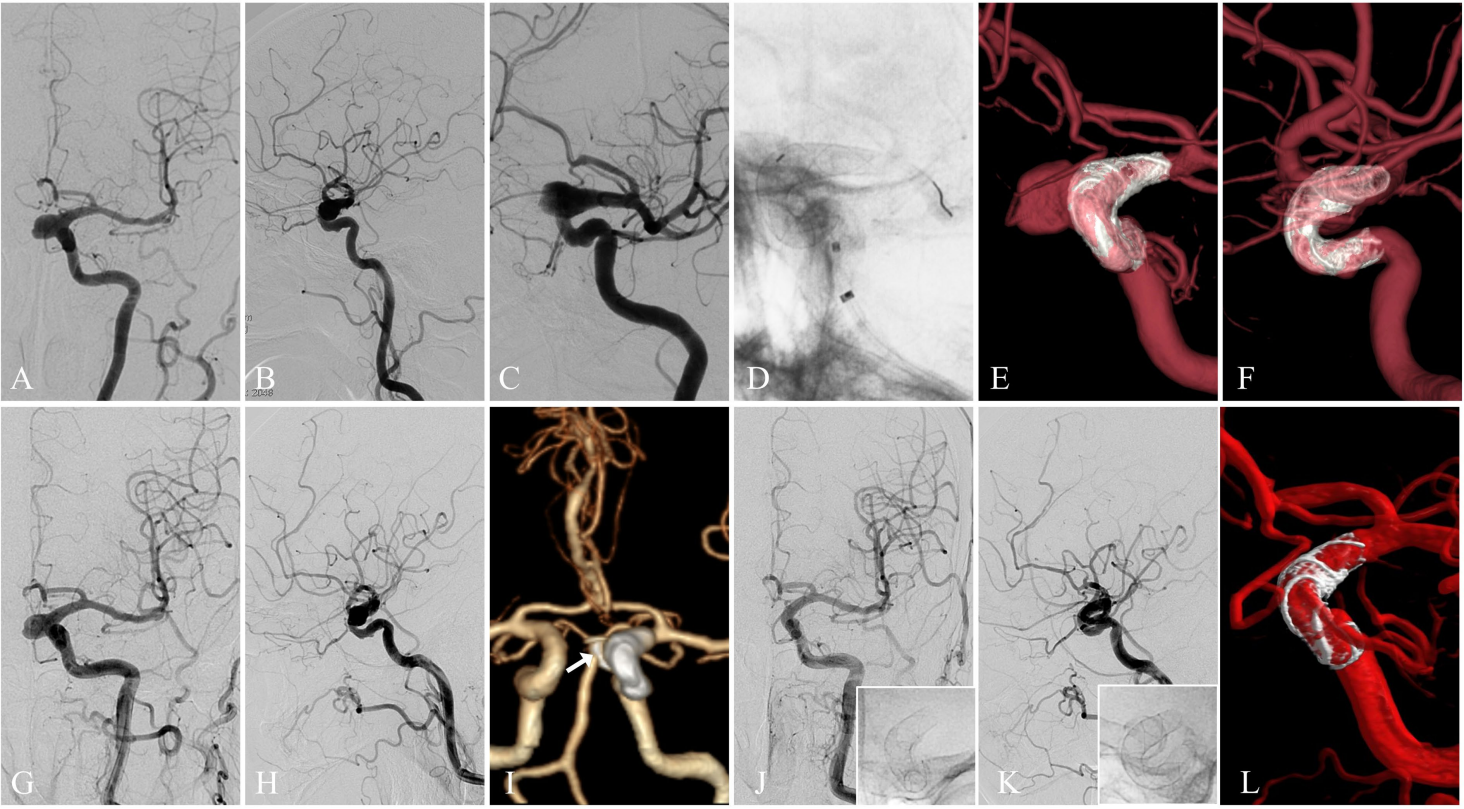
Angiographic images of a 69-year-old woman with a wide-neck aneurysm in the left ophthalmic ICA incidentally found after dizziness. **(A)** A-P and **(B)** lateral view of an aneurysm in the left ophthalmic ICA. **(C)** Intraprocedural working view of the aneurysm after TPFD (5 × 25 mm) deployment. **(D)** Fluoroscopic image of the TPFD. **(E)** Distal and **(F)** proximal apposition of TPFD confirmed by dual-volume 3D reconstruction. **(G)** A-P and **(H)** lateral view of incomplete occlusion of the aneurysm at the 6-month FU. **(I)** CTA image of the residual neck (arrowhead) of the aneurysm at the 12-month FU. **(J)** A-P and **(K)** lateral view of complete occlusion of the aneurysm at the 28-month FU. **(L)** Dual-volume 3D reconstruction at the 28-month FU.

At the 6-month FU, complete occlusion (OKM D) was achieved in 6 patients (60%), and residual aneurysm (OKM A/B) in 4 patients (40%). At the 1-year FU, complete occlusion (RROC I) was present in 8 patients (80%), residual neck (RROC II) in 1 patient (10%), and residual aneurysm (RROC III) in 1 patient (10%). Adequate occlusion improved from 60 to 90% (Figure 2).

The mean duration of the last FU was 29.40 ± 0.97 months (range, 28–31 months). Complete occlusion was achieved in 9 patients (90%), and residual neck in 1 (10%), yielding an adequate occlusion rate of 100%. No in-stent stenosis or occlusion was observed in any patient. All patients had a modified Rankin Scale (mRS) score of 0, indicating favorable clinical outcomes without ipsilateral strokes or device-related adverse events.

## Discussion

Flow diversion has fundamentally transformed the endovascular management of IAs, particularly for large, giant, dissecting, recurrent, and otherwise complex lesions poorly amenable to conventional coil embolization. The TFD has been increasingly adopted in clinical practice, with accumulating evidence supporting its safety and efficacy across diverse aneurysm morphologies and anatomical locations (Table 3).

**Table 3:** Published data on the Tubridge flow diverter in the treatment of intracranial aneurysms.

Author	Year	Study design	Patients		Aneurysm location	Aneurysm size (mm)	Aneurysm type	Aneurysm state	With coils	Technical success	Periprocedural		Angiographic follow-up					Clinical follow-up	
											Complication	Mortality	Rate	Month	Complete occlusion	Adquete occlusion	In-stent stenosis	Month	Favorable outcome
Zhou (14)	2014	Prospective single-center	28		ICA	Large to giant mean 21.6 (11.3–44)	Saccular	Unruptured recurrent	64.3%	97%	0	0	89.3%	Mean 9.9 (5–24)	72%	96%	0	Mean 19.0 (6–30)	100%
Jing (31)	2016	Prospective single-center	6		ICA	Large to giant mean 18.15 (12.15–25.61)	Saccular Dissecting	Unruptured	100%	100%	0	0	100%	Mean 8.5 (5–12)	66.7%	83.3%	NA	NA	NA
Fang (32)	2017	Prospective single-center	6		VA	Large (10.6–22.7)	Dissecting	Unruptured Recurrent	50%	100%	0	0	100%	Median 26.0 (18.5, 37.5)	83.3%	100%	16.7%	Median 36.5 (26.0, 44.5)	100%
Zhang (29)	2017	Prospective single-center	8		ICA, VA	Large to giant mean 16.7 (11.4–34.0)	Saccular Dissecting	Recurrent	25%	100%	12.5%	0	87.5%	Mean 16.9 (7–36)	71.4%	100%	14.3%	Mean 16.9 (7–36)	100%
Liu (9)	2018	Prospective multi-center RCT	82		ICA, VA	Large to giant median 18.0 (13.14, 26.0)	Saccular	Unruptured Recurrent	NA	96.34%	13.41%	3.66%	89.0%	6	75.34%	NA	5.48%	NA	NA
Liang (16)	2020	Prospective single-center	8		Complex MCA	Small to giant 11.8 ± 6.8	Saccular Fusiform	Unruptured Ruptured Recurrent	12.5%	100%	0	0	100%	7.5 ± 4.0	37.5%	50%	37.5%	11.3 ± 3.6	100%
Jia (33)	2020	Retrospective single-center	7		Cavernous-ICA	Large mean 18.3 (11.1–23.6)	NA	Unruptured	100%	100%	0	0	100%	57.5 ± 16.7 (6–69)	71.4%	100%	ISO 14.3%	73.32 ± 3.6 (66–78)	100%
Li (15)	2022	Retrospective single-center	23*		ICA, VA	Small to large	Saccular Dissecting	Unruptured	15.2%	96.3%	8.7%	0	78.3%	6–22	85.2%	88.9%	NA	6–22	100%
Wang (17)	2022	Retrospective single-center	13*		VBJ BA Trunk BA Tip	Medium to giant 14.6 ± 5.9 (5.8–27.4)	Dissecting Fusiform Saccular	Unruptured Recurrent	53.8%	100%	15.4%	7.7%	92.3%	8.17 ± 2.25 (5–12)	58.3%	91.7%	NA	8.17 ± 2.25 (5–12)	92.3%
Cai (18)	2022	Retrospective multi-center	53		ICA, VA, BA	Medium to giant	Saccular	Unruptured	90.6%	100%	3.77%	0	79.25%	9.1 ± 4.4	85.71%	95.24%	ISS 2.38% ISO 7.14%	NA	NA
Shi (19)	2022	Retrospective single-center comparative	43 TFD alone		ICA, VA, BA, PICA, Distal	8.7 ± 8.8	Saccular Dissecting Fusiform	Unruptured Ruptured	0	100%	8.2%	0	65.3%	8.7 ± 7.2	43.8%	46.9%	9.4%	8.7 ± 7.2	95.9%
			50 TFD with coil			12.9 ± 8.6			100%		10.9%		72.7%	11.3 ± 8.2	80.0%	97.5%	12.5%	11.3 ± 8.2	92.7%
Xie (28)	2023	Retrospective single-center comparative	57	39	ICA, MCA	Small 3.68 ± 0.82	Saccular Dissecting Fusiform	Unruptured	2.56%	100%	15.38%	0	94.9%	6.80 ± 1.70	88.46%	100%	2.56%	6.80 ± 1.70	100%
				18		Medium 7.61 ± 1.64			16.7%		0		94.4%	8.60 ± 1.30	81.82%	100%	0	8.60 ± 1.30	100%
Feng (34)	2023	Retrospective single-center	8		ICA	Small	BBA	Ruptured	100%	100%	0	0	100%	6–12	100%	100%	NA	6–12	100%
Xie (23)	2023	Retrospective single-center	23		VBA	Mean length 15.14 Mean width 9.14	Dissecting	Unruptured	8.7%	100%	13.04%	0	100%	Mean 6.04	78.26%	91.30%	4.35%	6.04	100%
Jin (24)	2023	Retrospective single-center	52		Posterior circulation	Small to giant 11.8 ± 7.0	Saccular Fusiform	Unruptured	13.5%	100%	9.6%	NA	76.9%	Mean 9.4 (6–24)	75%	NA	ISO 1.92%	26.4 ± 7.1	96.2%
Li (20)	2024	Retrospective single-center	85		ICA, VA, BA, Distal	Small to giant 8.7 ± 6.1 (2–30)	Saccular Dissecting Fusiform	Unruptured Ruptured	29.4%	100%	3.5%	0	78.8%	15.3 ± 5.6 (3–36)	83.6%	97.0%	7.5%	15.3 ± 5.6 (3–36)	100%
Liang (21)	2024	Retrospective multi-center	82		VA, BA, PCA	Median 8.00 (6.15, 11.38)	Saccular Dissecting Blister Fusiform	Unruptured Ruptured	14.6%	NA	15.9%	NA	NA	Median 7.0 (5.5, 13.0)	NA	72.9%	12.3%	Median 19.0 (6.25, 32.75)	87.5%
Huang (13)	2024	Prospective multi-center	290		ICA, VBA, Distal	Median 4.40 (3.10, 7.33)	Saccular non-saccular	Unruptured	14.1%	95.2%	2.8%	0	100%	Median 9.0 (6.0, 15.0)	75.5%	86.6%	16.6%	Median 18.0 (17.0, 19.0)	98.3%
Li (22)	2024	Prospective multi-center	200		ICA, VA, MCA	Small to giant 7.35 ± 5.73	Saccular Fusiform	Unruptured Ruptured	19.58%	99%	NA	NA	87.5%	12	79%	88.3%	3.6%	12	95.5%
Wan (30)	2025	Retrospective single-center	8*		ICA tandem	Small to large 5.29 ± 2.26 (2.7–11.3)	Saccular Fusiform	Unruptured	62.5%	100%	25%	0	100%	15.5 ± 13.0 (3–34)	76.5%	76.5%	12.5%	NA	100%
Duan (35)	2025	Retrospective single-center	25		MCA	Median 17.3 (4.2–27)	Dissecting	Unruptured Ruptured Recurrent	40%	100%	NA	0	100%	Median 13.0	56%	88%	16%	11–36	96%
Yuan (25)	2025	Retrospective single-center	144		Anterior & posterior circulation	Small to giant	Saccular Dissecting Fusiform	Unruptured Ruptured	52.1%	100%	16%	1.4%	66.0%	10.4 ± 8.7	84.6%	94.2%	4.2%	16.9 ± 10.1	96.6%
Juan (27)	2025	Retrospective single-center	72		ICA, MCA, VA	Small-giant median 4.20 (3.20, 7.68)	Saccular Dissecting Fusiform BBA	Unruptured Recurrent	37.3%	98.63%	8.33%	0	73.6%	Median 5.2 (3.45, 8.88)	71.43%	78.57%	32.73%	Median 13.5	97.01%
Huang (26)	2025	Retrospective single-center	12*		BA trunk, BA tip	Small-large 12.0 ± 6.1	NA	Unruptured Recurrent	25%	100%	16.7%	0	100%	13.4 ± 4.5	50%	83.3%	NA	Mean 12.9	100%

Data are number, %, median (interquartile range), mean (range), or mean ± SD.

BA, basilar artery; BBA, blood blister-like aneurysm; ICA, internal carotid artery; ISS, in-stent stenosis; ISO, in-stent occlusion; MCA, middle cerebral artery; PCA, posterior cerebral artery; PICA, posterior inferior cerebellar artery; RCT, random control trial; TFD, Tubridge flow diverter; VA, vertebral artery; VBA, vertebrobasilar aneurysm; VBJ, vertebrobasilar junction; ^*^TFD data was extracted from the mixed data of TFD and Pipeline.

To our knowledge, the present study is the first clinical report evaluating the feasibility, safety, and efficacy of the novel TPFD for IAs. As an upgraded iteration of TFD, TPFD incorporates design improvements intended to enhance deliverability, radiopacity, wall apposition, and hemodynamic remodeling while reducing thrombogenicity. In this prospective single-center cohort of 10 consecutive patients, we demonstrated 100% technical success without perioperative morbidity and mortality, progressive and durable aneurysm occlusion, and favorable clinical outcomes. These preliminary clinical data established the foundational evidence for TPFD and validated its potential as a safe and effective next-generation flow diverter in treating IAs.

### Technical success and procedural feasibility

Published literature on TFD consistently documented high technical success rates, ranging from 95.2 to 100%. In a large prospective multicenter study by Huang et al. (13) including 290 patients, the technical success rate was 95.2%, with failures mainly attributed to poor apposition, re-deployment requirements, FD migration or shortening. Liu et al. (9) reported a technical success rate of 96.34% in a randomized controlled trial of 82 patients with large and giant aneurysms, with difficulties including distal artery catheterization and device delivery. Moreover, rare instances of technical hurdles included mid-stent opening and distal FD herniation into the aneurysm sac had also been reported (14, 15). Adjunctive maneuvers such as microguidewire massage manipulation, balloon angioplasty, or rescue stenting were frequently required to achieve technical success, especially in complex anatomies (16–22). In our TPFD cohort, technical success was 100%. Although two patients required adjunctive stenting for malapposition, no procedural failure or conversion to alternative treatment occurred. Whether this favorable performance may be related to key design enhancements, including a smaller-profile 0.027-inch microcatheter, full-body radiopacity, and optimized flared ends, remains to be determined. Direct comparative studies will be necessary to evaluate whether these iterative modifications translate into measurable clinical advantages over the original TFD.

### Perioperative safety and complications

Perioperative safety is a critical determinant of flow diverter utility. Pooled data from available TFD studies show overall complication rates ranging from 0 to 25%, with mortality rates generally 0–7.7%. Ischemic events, including thromboembolism, branch occlusion, small infarcts, and in-stent thrombosis, represented the most common category (9, 13, 18, 19, 23–27). Li et al. (20) reported a 3.5% perioperative complication rate mainly consisting of transient in-stent thrombosis, small arteries occlusion and multiple cerebral infarctions. Xie et al. (28) observed a 15.38% rate of new mild cerebral infarctions, primarily in patients with small aneurysms. Hemorrhagic complications, including intraoperative or delayed aneurysm rupture, and postoperative intracerebral hemorrhage (ICH), were less frequent but carried higher severity (9, 13, 19, 25). Liu et al. (9) reported a 13.41% combined ischemic and hemorrhagic complication rate and a 3.66% mortality rate, mostly related to vessel injury or delayed aneurysm rupture. Other adverse events or complications included vasospasm, dissection, and puncture-site complications, most of which were reversible or clinically silent (25, 27, 29, 30). Notably, several studies reported zero perioperative complications and mortality, supporting the favorable safety of TFD in treating IAs (14, 16, 31–34). In our initial TPFD experience, no perioperative complications, permanent neurological deficits, or deaths occurred. Only one patient experienced transient postoperative headache as a result of mass effect, which resolved spontaneously. This favorable result might be attributable to small sample size, careful patient selection, as well as timely and effective management of intraoperative adverse events.

### Angiographic outcomes

Angiographic outcomes following TFD treatment reflected the natural course of flow diversion, with gradual intra-aneurysmal thrombosis and endothelialization over months. Complete occlusion rates in published studies varied widely, from 37.5 to 100%, which was primarily determined by the location and morphology of the aneurysms. Moreover, adequate occlusion was achieved with greater consistency, with rates ranging from 50 to 100% in most cohorts. Zhou et al. (14) achieved 72% complete occlusion and 96% adequate occlusion at a mean FU of 9.9 months. Liang et al. (16) reported relatively lower complete occlusion (37.5%) and adequate occlusion (50%) in complex middle cerebral artery (MCA) aneurysms, likely related to the inherent properties of dissecting MCA aneurysms and small sample size. In large-sample studies, Li et al. (20) reported 83.6% complete occlusion and 97.0% adequate occlusion in 85 patients, while Yuan et al. (25) showed 84.6% complete occlusion and 94.2% adequate occlusion in 144 patients.

In-stent stenosis (ISS) was reported in 0 to 32.73% of patients. Most cases were asymptomatic and managed conservatively. Huang et al. (13) noted a 16.6% overall ISS rate but only 1.7% symptomatic stenosis, indicating favorable clinical tolerance. Among studies documenting in-stent occlusion (ISO), Cai et al. (18) reported an ISO rate of 7.14% in a multicenter retrospective series of 53 patients. This finding indicated that overt luminal occlusion occurred in a small but notable subset of patients.

In our TPFD series, complete occlusion rates improved progressively over time: 60% at 6 months, 80% at 1 year, and 90% at the last FU, with 90 and 100% adequate occlusion rate at 1-year and last FU, respectively. Moreover, no ISS or ISO was detected. This observation is encouraging, though whether it can be specifically attributed to the BlueSilk Surface^®^ technology or to satisfactory device apposition requires confirmation in larger controlled studies. The only case of residual neck (10%) at the last FU might be attributed to the ophthalmic artery arising from the base of the aneurysm neck, which limited complete occlusion due to persistent hemodynamic inflow. However, the absence of residual aneurysm lumen revealed that TPFD was sufficient to prevent aneurysm growth or rupture.

### Clinical outcomes

Clinical outcomes with favorable outcomes (mRS 0–2) in published TFD studies ranged from 87.5 to 100%. Huang et al. (13) reported 98.3% favorable outcomes in 290 patients, and Jin et al. (24) achieved 96.2% in 52 patients. Transient neurological worsening, such as headache or mild oculomotor nerve palsy related to mass effect in large/giant aneurysms, was occasionally described but resolved without permanent deficit. In our cohort, all 10 patients achieved favorable clinical outcome (mRS = 0) at last FU, indicating full functional recovery and no procedure-related disability.

### Treatment strategy and adjunctive coiling

The use of adjunctive coiling with TFD varied widely across studies, from 0 to 100%, based on aneurysm size, morphology, rupture status, and operator preference. Adjunctive coiling with TFD was often used for large, giant, ruptured, or recurrent aneurysms to accelerate intra-aneurysmal thrombosis. Jing et al. (31) and Feng et al. (34) used adjunctive coiling in 100% of cases, mostly for large or ruptured blister aneurysms. Shi et al. (19) directly compared TFD alone versus TFD with coils, showing higher complete and adequate occlusion rates in the combined group, albeit with a slightly higher complication rate. In our study, 80% of patients were treated with TFD alone and 20% with TFD plus coils, consistent with the trend that simple aneurysms could be effectively managed with flow diversion alone, while complex or large lesions might benefit from adjunctive coiling to accelerate thrombosis.

## Limitations

This study had several important limitations. First, it was a single-center design with a small sample size (10 patients), limiting generalizability. Second, although mid-term FU was favorable, longer-term surveillance exceeding 3–5 years is needed to assess delayed complications such as in-stent stenosis or delayed aneurysm occlusion. Third, no control group was included for direct comparison with the original TFD or other FD devices. Fourth, all aneurysms were located in the anterior circulation (ICA), and no posterior circulation or ruptured aneurysms were included. Additionally, the potential for CTA-related metal artifacts to overestimate residual aneurysm filling should be considered when interpreting long-term occlusion rates. Future large-scale, prospective, multicenter studies with long-term FU are warranted to validate these findings across a broader spectrum of aneurysms.

## Conclusion

In summary, this prospective single-center preliminary experience in 10 patients suggests that the TPFD, an upgraded iteration of the TFD, is technically feasible, safe, and effective for the endovascular treatment of unruptured ICA aneurysms. These early clinical data provide foundational evidence for the TPFD. However, given the small sample size and single-center design, further large-scale, multicenter studies with long-term FU are warranted to validate its safety and efficacy as a next-generation flow diverter.

## Data Availability

The original contributions presented in the study are included in the article/supplementary material, further inquiries can be directed to the corresponding authors.
